# Investigating the Randomness of Passengers’ Seating Behavior in Suburban Trains

**DOI:** 10.3390/e21060600

**Published:** 2019-06-17

**Authors:** Jakob Schöttl, Michael J. Seitz, Gerta Köster

**Affiliations:** Department of Computer Science and Mathematics, Munich University of Applied Sciences, Lothstr. 34, 80335 Munich, Germany

**Keywords:** randomness, entropy, pedestrian behavior, traffic models, traffic and crowd dynamics, agent based models, seating behavior, field observation

## Abstract

In pedestrian dynamics, individual-based models serve to simulate the behavior of crowds so that evacuation times and crowd densities can be estimated or the efficiency of public transportation optimized. Often, train systems are investigated where seat choice may have a great impact on capacity utilization, especially when passengers get in each other’s way. Therefore, it is useful to reproduce passengers’ behavior inside trains. However, there is surprisingly little research on the subject. Do passengers distribute evenly as it is most often assumed in simulation models and as one would expect from a system that obeys the laws of thermodynamics? Conversely, is there a higher degree of order? To answer these questions, we collect data on seating behavior in Munich’s suburban trains and analyze it. Clear preferences are revealed that contradict the former assumption of a uniform distribution. We subsequently introduce a model that matches the probability distributions we observed. We demonstrate the applicability of our model and present a qualitative validation with a simulation example. The model’s implementation is part of the free and open-source Vadere simulation framework for pedestrian dynamics and thus available for further studies. The model can be used as one component in larger systems for the simulation of public transport.

## 1. Introduction

Pedestrian dynamics span a wide field of research from empirical studies to mathematical modeling [1]. Microscopic, that is, individual-based models of human locomotion are used to simulate crowd motion and study emergent behavior (see [2,3,4] for overviews). The goal is often to improve safety by estimating evacuation times and crowd densities [5,6,7,8,9] while also optimizing efficiency in public transport. For example, passenger exchange times are estimated by having virtual pedestrians leave and board trains [10]. Thus, it seems necessary to correctly reproduce passengers’ behavior inside a train. It is common practice to assume a uniform distribution of passengers in the train (see, e.g., [11]) or at least on the seats, partly for convenience but mostly because there is no accepted empirical evidence on the subject. However, we argue that the positions of passengers has an influence on passenger exchange times. For example, window seats are a little further away from the exits than aisle seats. More importantly, the seating positions determine how much passengers get in each other’s way. Passengers sitting directly across each other cannot get up at the same time, while passengers sitting diagonally across can. This may cost precious seconds during extremely short inner city stops. In addition, many simulation models are inspired by entropy-based multiple particle systems [12]. However, this entails an implicit assumption on the entropy of human seating behavior. In this study, we aimed at validating or falsifying this assumption by observing empiric distributions.

Our second goal was to build a simulation model that matches our observations. The literature on simulation models is extensive. Simulation software can cope with common scenarios including gates, queues [13,14,15,16,17], stairs, and multiple floors [14,15,18,19,20,21]. However, validated software that models more complex social interactions, such as helping, is still rare. While well-designed frameworks that allow, in principle, to add on or easily build behavioral models exist (e.g., [22]), the behaviors that are implemented usually lack an empirical foundation [1]. There are few exceptions [11]. At this point, we are not aware of any specific simulation models for trains but there are several related publications on boarding schemes for airplanes [23,24,25].

Empirical studies are the only way to validate models of human behavior that aim at quantitatively predicting phenomena. An extensive survey of controlled experiments and field observations published in peer-reviewed scientific journals can be found in [1]. The research is ongoing. Interestingly, some valuable information on seating behavior that we found, using the terms “choice of seating” and “preferred seating” combined with “public transport” and “trains” in English and German on Google Scholar, was provided in journals that target practitioners where, unfortunately, it is likely to be overlooked by academic research. There are studies on the degree of capacity utilization [26], baggage on trains [26,27,28], passenger exchange times [29,30], and train interior design [28]. Studies on seating layouts and passengers’ seating behavior in trains are discussed by several authors. For example, New York commuters who have to sit close to other passengers experience adverse reactions [31], Australian passengers try to “retreat into their personal ‘bubble’”, for example, by acquiring a seat as quickly as possible [32]. Both observations fit well with the more general concept of personal space which persons try to safeguard [33]. Preferred facing directions on the Washington Metro are investigated in [34], and preferred seat choices on German inter-city trains in [35]. A survey on passengers’ valuation of seating layouts in British public transportation is presented in [36]. Other research focuses on the inflow process when people enter a room [37,38,39,40]. The participants could not sit down in the experiments of these studies, but some aspects are related to our work. Regarding the choice of wagon, we did not find any quantitative data during our literature research. Various authors [30,37,38,41] look at the distribution of passengers on the platform and the waiting process. On a qualitative level, passengers develop habits as a means to build a private zone [32]. Experts of Munich public transport (MVV) with whom we discussed our project emphasize the role of the local layout of the start and end stations of passenger journeys, which is corroborated in [30].

Overall, prior reports seem to suggest that there are a number of influences on seating behavior that foster preferences in seat choices. However, there are few results on people’s seating behavior on trains that can be used to build a simulation model. Our study also aimed at closing this gap. In the following sections, we present empirical data on how single passengers sit down in relation to other passengers. We built a seating model from our observations and implemented it within the open-source Vadereisimulation framework. See [42,43,44] and the website www.vadere.org for an introduction to the software. Next, we verified the model’s implementation by comparing simulated data to data from a field observation. Then, we qualitatively validated the model by demonstrating that the train fills up in a visually realistic manner. Both methods are based on a simulation run with real passenger counts. We conclude with a short summary, a discussion, and inspiration for future work in this field.

## 2. Material and Methods

### 2.1. Field Observation: Data Collection

**Figure 1:**
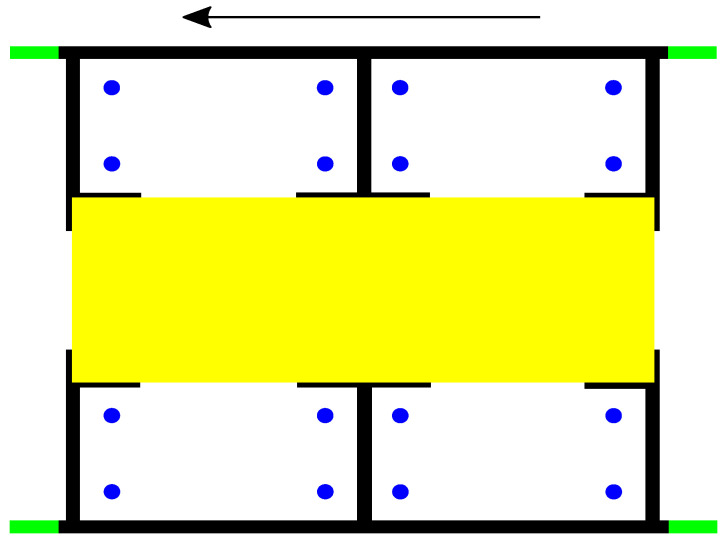
Compartment with four seat groups, with four seats each. The black arrow on the top denotes the train’s driving direction. The blue dots mark the seats and the yellow area in the middle is the aisle connecting the entrance areas. The green bars are parts of the doors.Plan of a S-Bahn train compartment.

The first author used his own smart phone app, on a Nexus 6 with Android OS, over a period of several weeks, to collect fully anonymous passenger data on his train ride to and from Munich University of Applied Sciences in Munich’s suburban trains (S-Bahn). We chose to write an app not only for convenience but also to mitigate the risk for a personal bias caused by the single observer. Predefined choices forced him to be systematic. The app also allowed him to be quick in order to not miss behavioral events. He observed single compartments (see Figure 1) during train rides, logging relevant events such as sitting down, leaving, and placement of baggage. While he also collected information on the estimated age group and gender of passengers, we did not, in this contribution, investigate the effect on seating preferences. For more details, especially on requirements for and testing of the app, we refer to [45].

### 2.2. Field Observation: Data Analysis

For data analysis, we used R, a language for statistical computing. We tested the R code for data processing and analysis with the R unit test frameworks RUnit and especially testthat [46]. The test code amounted to about 600 lines of test cases split into 8 source files. Both data and code are open-source and hosted at GitHub: https://github.com/schoettl/seating-data. The data are licensed under the Public Domain Dedication and License (PDDL) while the code is licensed under the MIT License.

For all observed preferences, binomial tests were conducted using the exact binomial test binom.test from R’s stats package to see whether they are statistically significant. The significance level was set to α=0.05.

## 3. Results

### 3.1. Seating Preferences

**Figure 2:**
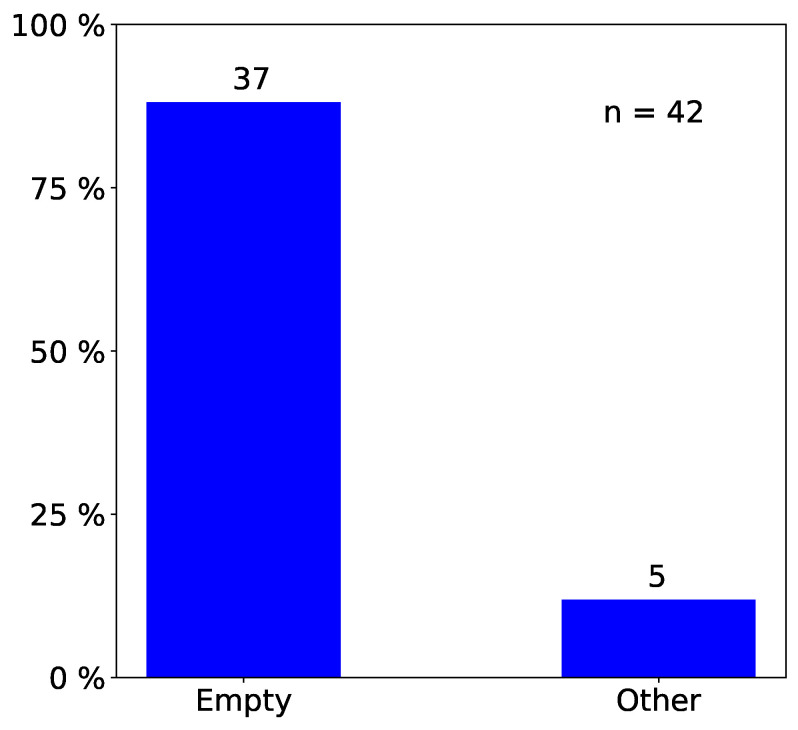
Observed frequency of seat choices: There is a strong preference for empty seat groups over seat groups where another passenger is already seated. We only consider data where both empty and preoccupied seat groups are available (n=42).

**Figure 3:**
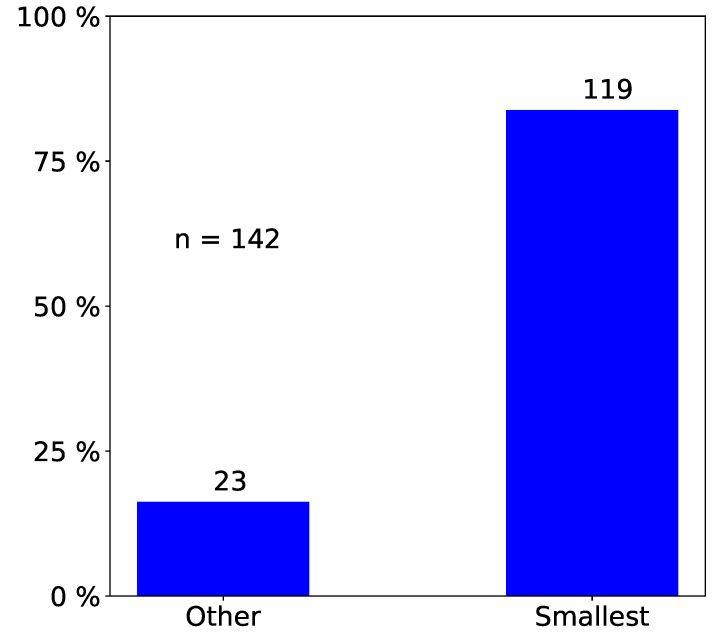
Observed frequency of seat choices: There is a strong preference for the seat group with the smallest number of occupants that are already seated. We only consider data where the occupancy of the seat groups differs (n=142).

**Figure 4:**
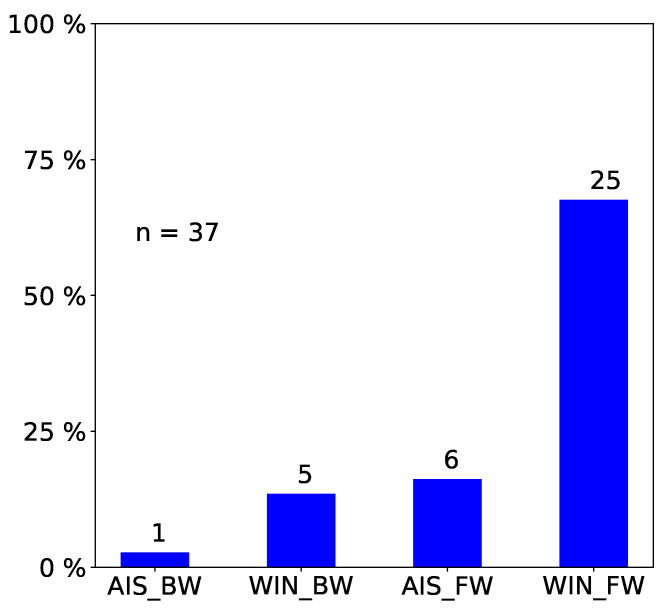
Observed frequency of seat choices: When a seat group is empty forward seats are chosen more often than backward seats, and window seats more often than aisle seats. AIS, aisle; WIN, window; FW, forward; BW, backward. We only consider incidents where a passenger sits down in an empty seat group (n=37).

**Figure 5:**
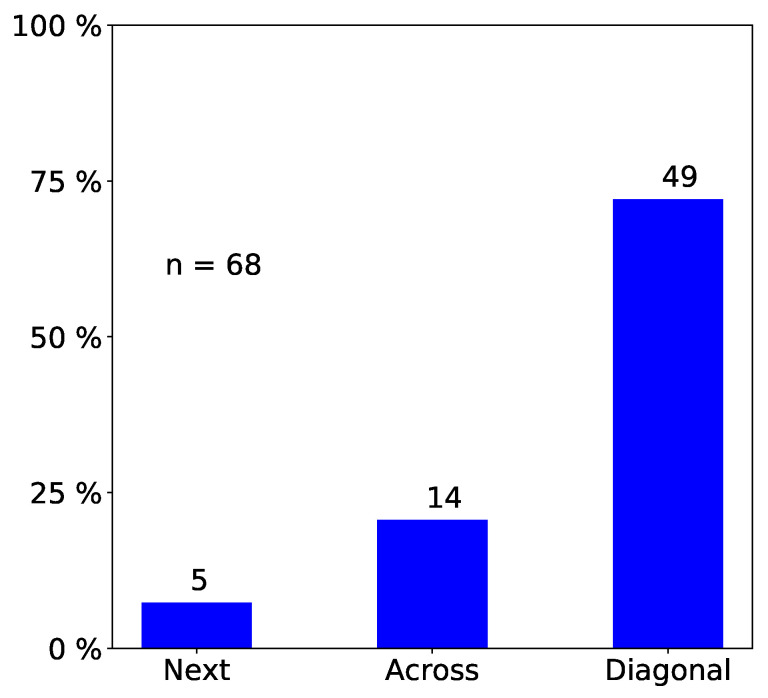
Observed frequency of seat choices: When one seat is already taken, the choice diagonally across is chosen most often. Next, neighbor seat; Across, seat directly across; Diagonal, seat diagonally across. We only consider incidents where a passenger sits down in a seat group where exactly one seat is taken (n=68).

We only report statistically significant results. The total number of observed incidents depends on what we investigated. For easy reading, we state it in each figure with “n=totalnumberofobservedincidents”. Passengers prefer empty seat groups or, more generally, they prefer the seat group with the fewest occupants (see Figure 2 and Figure 3). Apparently, passengers try to maximize the distance to other passengers, which is in line with the safeguarding of personal space described in psychological studies [32,33] and in particular with the discomfort experienced when sitting close to others reported in [31]. When a seat group is empty, forward seats are chosen more often than backward seats, and window seats more often than aisle seats (Figure 4). When one seat is already taken, the choice diagonally across wins (Figure 5).

During his train rides, the first author observed that passengers often choose one of the compartments next to their entrance area. In the same anecdotal way, he observed that, once passengers have chosen a direction, they rather proceed to the next compartment instead of turning around and going back, and, when passengers choose a compartment in a different train section, they tend to directly walk to this section. Systematic surveys on this behavior would be interesting follow-up studies.

### 3.2. The Seating Model: Algorithm and Test

For our seating model, we used a combination of cognitive heuristics [19] and random assignment according to the empirical distributions. That is, we let the simulated agents make decisions depending on the situation they are confronted with when entering the train: they choose their seats with a probability that matches the empirical preferences.

**Figure 6:**
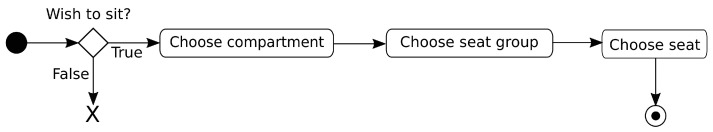
The seating algorithm: Passengers pick their seat according to the empirical preferences. They choose a compartment close to where they enter, following a normal distribution.

The algorithm’s first decision is whether the passenger wants to sit at all. For this work, we assumed that all passengers wish to sit. According to the findings in [32], this may be a reasonable assumption for most passengers, since a seat offers a protected space. For simplicity, we did not consider reasons to keep standing, for example when a passenger needs to exit in a very crowded situation [32]. The rest of the algorithm consists of three steps: choosing a compartment, choosing a seat group, and choosing a seat therein (see Figure 6). We used a truncated normal distribution to assign each agent a compartment where they sit down. For the choices of seat group and seat, we used the relative frequencies from our field observations that were statistically significant. Otherwise, we used a uniform distribution. The chosen seat was then assigned as target in the floor field-based navigation of the Optimal Steps Model [42,43] in the Vadereisimulation framework [44]. That is, agents find their paths to their seats by optimizing, in each step they take, a utility function that is coded in the floor-field. The closer they are to the seat, the higher is their utility. Distance to the seat is not measured through Euclidean distance but by computing geodesics [4,47] so that obstacles are skirted. Other agents cause a dip in utility so that agents keep a distance to each other. Stepping on other agents is completely excluded. Alternatives would be the Behavioral Heuristics Model [48], the Gradient Navigation Model [49] or any other microscopic locomotion model that can handle the fine spatial resolution of the geometry of Munich’s ET-423 S-Bahn train. A coarse spatial resolution, as common in most cellular automata, may lead to passengers getting stuck unless one forgoes the paradigm of one agent per cell [50]. Some special cases, such as a full compartment or that another passenger snatches one’s chosen seat, have to be handled. For this and for parameter choices in the Optimal Steps Model, we refer to [45].

**Figure 7:**
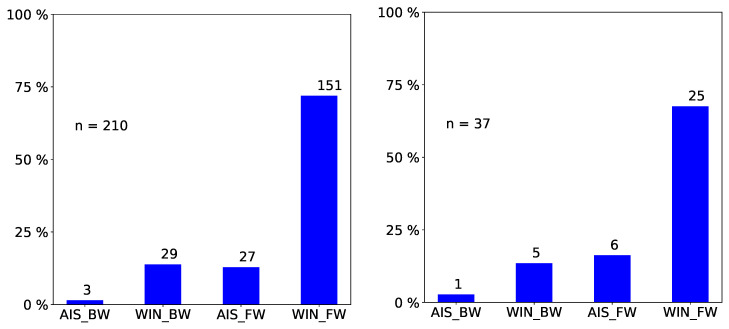
Program verification: Comparison of simulation outcome (first) to empirical data (second) for choice of seat in an empty seat group.

**Figure 8:**
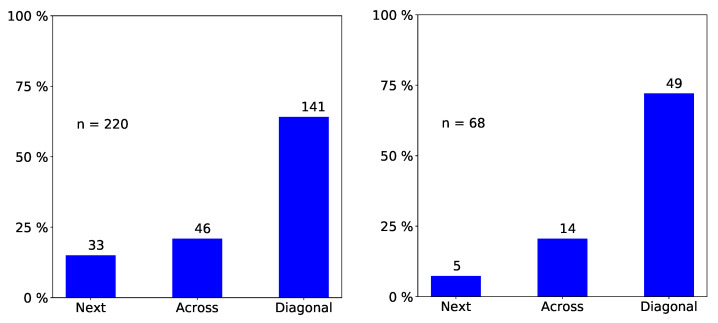
Program verification: Comparison of simulation outcome (first) empirical data (second) for choice of seat within a seat group when one seat is already taken.

We conducted simulation runs to test our implementation. Since we used the statistical distribution as an input, correct reproduction of the empirical data amounted to verification. Figure 7 and Figure 8 are examples for the series of comparisons. Further data comparisons can be found in the first author’s master thesis [45]. For standard statistical tests ($\chi^{2}$ and Watson-$U^{2}$), the null hypothesis that the two data sets have the same distribution was not rejected at the 5% level.

**Figure 9:**
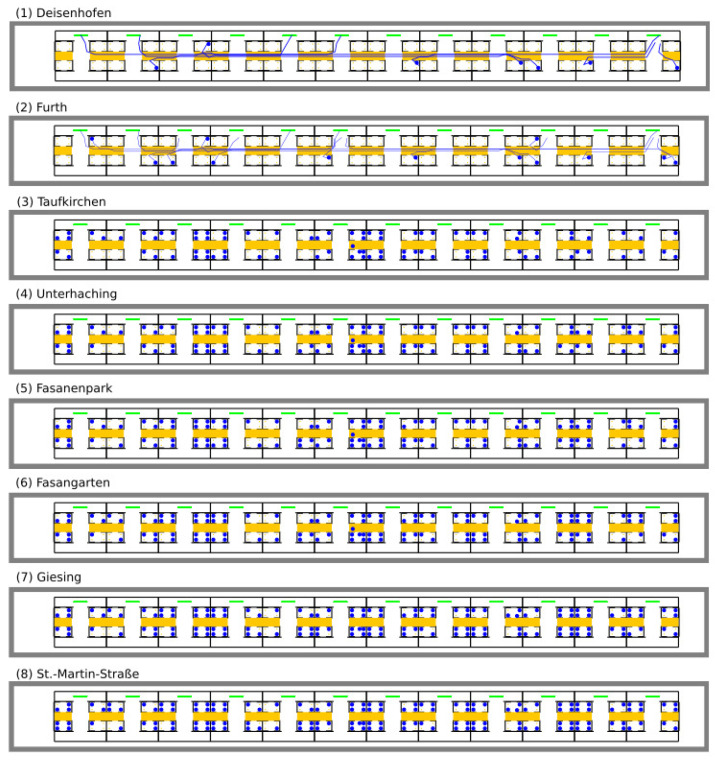
Visualization of a simulation run. At each virtual train station, new agents board the train through doors marked in green. No passengers alight, so that the train gets fuller and fuller. At the top of each line, the name of the train station is stated, e.g., Deisenhofen.

We achieved a qualitative validation by visually examining the plausibility of simulation runs (Figure 9). At each virtual train station, new agents board the train through doors marked in green. No passengers alight, so that the train gets fuller and fuller.

## 4. Conclusions

We designed a study on passengers’ seating behavior on trains, developed a mobile app to support data logging, collected data, and processed and analyzed the data with respect to the choice of seating. Thus, we gained quantitative distributions on where passengers prefer to sit in a compartment and in a seat group. We revealed clear preferences, and thus a degree of order compared to the uniform distribution that is often assumed for convenience. Based on the results of the data analysis, we designed a model for seating behavior and implemented it in the open-source crowd simulation software Vaderei, where it is available for further studies. The model can easily be included in larger simulation systems for public transport to make predictions of important control quantities such as flow, density or passenger exchange times more realistic. Our results are in line with psychological findings, namely the fact that people like to safeguard their personal space, keeping a distance from others. In the context of entropy, we interpret the psychological factors as external influences, or energy, that introduce a certain degree of order to the transportation system, and thus reduce the entropy of this open system. In summary, we were able to fill a gap in pedestrian dynamics: an empirically substantiated seating model.

For many applications, the model could be refined: psychological factors, such as the influence of gender or age on the seat choice, or the behavior of groups, as well as the reasons behind the choice of compartments, would be interesting follow-up studies. An empirical study and model of the deboarding process would complement this work and allow better predictions of passenger exchange times. Quantitative studies on wagon choices would not only advance modeling but directly help to fill trains in a safer and more efficient way.
